# High sustained viral response among HCV genotype 3 patients with advanced liver fibrosis: real-world data of HCV elimination program in Georgia

**DOI:** 10.1186/s13104-020-05173-4

**Published:** 2020-07-11

**Authors:** Maia Butsashvili, Lia Gvinjilia, George Kamkamidze, David Metreveli, Shorena Dvali, Tamar Rukhadze, Amiran Gamkrelidze, Muazzam Nasrullah, Shaun Shadaker, Juliette Morgan, Francisco Averhoff

**Affiliations:** 1 Clinic NEOLAB, 47 Tashkenti Street, Tbilisi, 0160 Georgia; 2 CDC Foundation, 9 Asatiani Street, Tbilisi, 0177 Georgia; 3 Medical Center Mrcheveli, 9 Al. Kazbegi Ave, 0160 Tbilisi, Georgia; 4 Infectious Diseases, AIDS and Clinical Immunology Research Center, 16 Al. Kazbegi Ave, 0160 Tbilisi, Georgia; 5 Clinic HEPA, 18/20 Ljubljana Street, Tbilisi, 0159 Georgia; 6 National Center for Disease Control and Public Health, 9 Asatiani Street, Tbilisi, 0177 Georgia; 7 Division of Viral Hepatitis, National Center for HIV/AIDS, Viral Hepatitis, STD and TB Prevention, CDC, 1600 Clifton Road, Atlanta, GA 30329-4027 USA; 8 Epidemiology, Informatics, Surveillance and Laboratory Branch (EISLB), Division of Global Health Protection (DGHP), Center for Global Health (CGH), CDC, 1600 Clifton Road, Atlanta, GA 30329-4027 USA

**Keywords:** HCV, SVR, Genotype 3, Fibrosis, Liver, Elimination

## Abstract

**Objective:**

In 2015, Georgia launched HCV elimination program. Initially, patients with advanced liver disease were treated with sofosbuvir-based regimen—the only DAA available for all genotypes. Purpose of the study was assessing real-world data of treatment outcome among patients with HCV GEN3 and advanced liver fibrosis with sofosbuvir-based regimens.

**Results:**

Totally 1525 genotype 3 patients were eligible for analysis; most (72.6%) were aged > 45 years, majority were males (95.1%), and all (100%) had advanced liver disease (F3 or F4 by METAVIR score based on elastography). Of those who received sofosbuvir/ribavirin (SOF/RBV) for 24 weeks, 79.3% achieved SVR, while 96.5% who received sofosbuvir/pegylated interferon/ribavirin (SOF/PEG/RBV) for 12 weeks achieved SVR (p < 0.01). Among patients with liver cirrhosis (defined as F4) overall cure rate was 85.7% as opposed to 96.4% for those with F3. Females were more likely to be cured (98.7% vs 89.7%; OR = 8.54). Patients aged 31–45 years had higher likelihood of achieving SVR compared to patients aged 46-60 years (95.7% vs 87.4%; OR = 0.32,). Independent predictors of SVR were treatment with SOF/PEG/RBV (aOR = 6.72) and lower fibrosis stage (F3) (aOR = 4.18). Real-world experience among HCV GEN3 patients with advanced liver fibrosis and treated by sofosbuvir regimen w/o PEGIFN, demonstrated overall high SVR rate.

## Introduction

The World Health Organization (WHO) estimates, globally 71 million people are living with chronic hepatitis C virus (HCV) infection, and 400,000 die annually, mostly from complications of cirrhosis and hepatocellular carcinoma [1]. Recently introduced direct-acting antivirals (DAAs) offer an opportunity for curing the vast majority of infected persons, which will reduce the transmission risk and prevalence of HCV in the population.

Georgia has a high burden of HCV infection; a 2015 national serosurvey found that an estimated 5.4% of adults are currently infected with HCV [2]. On April 28, 2015, Georgia launched the world’s first National HCV Elimination Program that included free of charge treatment with DAAs for all HCV infected persons [3]. The DAAs for the elimination program are donated by Gilead Sciences, and sofosbuvir was the first DAA available for the program. In the initial phase of the program, patients with moderate or severe liver disease were prioritized to receive treatment [3]. Cure rates for HCV infection (i.e., FSustained Virologic Response or SVR) varies depending on the genotype, degree of liver fibrosis, and the specific DAAs used [4–11]. HCV infected patients with genotype 3 are considered difficult to treat with SOF/RBV and SOF/PEG/RBV regimens, compared to other genotypes [5, 12, 13].

The HCV elimination treatment program in Georgia, with a large number of patients with genotype 3 patients offers a unique opportunity to study the outcomes among these hard to treat patients in a real-world setting. We aimed to study the real-world treatment outcomes among genotype 3 HCV infected patients with advanced disease treated with SOF/RBV and SOF/PEG/RBV regimens. Despite the fact that IFN-containing regimens are no longer standard of care in developed world, developing countries still use some of these regimens.

## Main text

### Methods

The Georgia National HCV Elimination Program collects data on enrolled patients’ pre-treatment, during treatment, and post treatment. Data collected includes socio-demographic information, clinical and laboratory data, and prescribed medications based on national guidelines upon enrolment. These data are collected using standardized protocols, and entered into a national treatment database, STOP-C, developed for the HCV elimination program. Data collected and stored in STOP-C includes HCV genotype and viral load, level of liver fibrosis, risk factors for HCV infection and treatment-related laboratory data, including SVR at week 12–24 after completion of treatment.

Data from April 28, 2015 through September 30, 2016 from STOP-C were analysed. Characteristics and outcomes of patients with genotype 3 were extracted. Only patients with advanced fibrosis (F3 or F4 by METAVIR score based on elastography) who had SOF-based regimens and valid SVR 12-24 results were included in the analysis. The treatment for patients with genotype 3 per national guidelines, was either sofosbuvir and ribavirin (SOF/RBV) for 24 weeks, or sofosbuvir, ribavirin and pegylated interferon (PEG IFN) 2a or 2b (SOF/PEG/RBV) for 12 weeks, depending on patient eligibility to receive IFN. SVRs were calculated using per-protocol as well as intent-to-treat (ITT) analysis. Per-protocol analysis included patients with complete SVR data and ITT analysis also included those who discontinued treatment. Treatment outcomes were analysed by degree of liver fibrosis (F4-cirrhosis vs F3-non-cirrhosis) and treatment regimen. Statistical software, SAS version 9.4, was used for data analysis. Bivariate associations between treatment outcome with different factors, such as treatment regimen, fibrosis stage, age, and gender were analysed using Chi square. Multivariate analysis with logistic regression was used to estimate odds ratios adjusted for age and gender (aORs) and define independent predictors of SVR.

### Results

A total of 1525 genotype 3 patients completed their SOF-based treatment and had an SVR result available for analysis (Table 1). The overall cure rate i.e., SVR for the genotype 3 patients was 90.1% (1374/1525). Among patients with liver cirrhosis (F4 by elastography) the overall cure rate was 85.7% (764/892) as compared to non-cirrhotic patients (F3 or F3/F4) where 610/633 (96.4%) achieved SVR. The SVR rate was significantly higher among those treated with SOF/INF/RBV containing regimen compared to those ineligibles for prescribing interferon-based treatment. Out of 959 patients receiving PEG IFN 2a or 2b with SOF and RBV for 12 weeks, 925 (96.5%) achieved SVR compared to 79.3% cure rate among those treated with SOF and RBV for 24 weeks (449 out of 566). The SVR rate in intent-to-treat analysis for IFN/SOF/RBV regimens was 76.9% and for SOF/RBV regimen—61.2%.

**Table 1:** Sustained virologic response (SVR) by age, gender and fibrosis stage (N = 1525), nationwide HCV elimination program, Georgia, April 28, 2015–September 30, 2016

	Total n (%) N = 1525	SVR achieved n (%) N = 1374	Unadjusted OR and 95% CI	Adjusted OR and 95% CI
Age group				
18–30	7	7 (100.00)	–	
31–45	411	393 (95.62)	1	
46–60	986	862 (87.42)	0.32 (0.19, 0.53)	
> 60	121	112 (92.56)	0.57 (0.25, 1.30)	
Gender				
Male	1450	1300 (89.66)	1	
Female	75	74 (98.67)	8.54 (1.18, 61.87)	
Fibrosis stage				
F4	892	764 (85.65)	1	1
F3 or F3/F4	633	610 (96.37)	4.44 (2.82, 7.01)	4.18 (2.64, 6.61)
Treatment regimen				
SOF/RBV	566	449 (79.33)	1	1
SOF/INF/RBV	959	925 (96.45)	7.09 (4.76, 10.56)	6.72 (4.49, 10.06)

By bivariate analysis, gender was significantly associated with SVR rate. Females (74/75 [98.7%] were more likely to be cured compared to males 1300/1450 [89.7%]; OR = 8.54, 95% CI 1.18–61.87). Patients aged 31–45 years had higher chance of achieving SVR (95.7% vs 87.4%) compared to patients aged 46–60 years (OR = 0.32, 95% CI 0.19–0.53).

Multivariate analysis showed that the independent predictors of achieving SVR were treatment regimen (patients treated with SOF/IFN/RBV combination were more likely to be cured–aOR = 6.72, 95% CI 4.49–10.06) and fibrosis stage (non-cirrhotic patients having higher chance of SVR–aOR = 4.18, 95% CI 2.64–6.61) (Table 1).

### Discussion

In this analysis of the real-world experience among HCV genotype 3 infected patients with advanced liver fibrosis treated with SOF containing regimen with or without pegylated interferon, we found patients achieved overall high SVR rates of > 90%. Higher SVR rate was observed among women. This finding is comparable to other studies [6, 14, 15] where SVR rate varied by gender with males having lower HCV cure rate. The factors account for this difference are not well understood. Patients with liver cirrhosis in our cohort achieved higher SVR rates with this “first generation” DAA compared to previous published reports that enrolled cirrhotic patients with HCV genotype 3 [13, 16]. Several studies demonstrated SVR rates of 60% to 70% among those receiving the SOF and RBV 24-week regimen [6, 16–19]. The VALENCE trial reported an overall SVR rate of 85% among genotype 3 infected patients receiving the 24-week SOF/RBV regimen [6]. In the VALENCE trial, SVR rates at 12-weeks post-treatment (SVR 12) were 91% for the non-cirrhotic group and 68% for the group of study participants with liver cirrhosis, and multivariate analysis identified the presence of liver cirrhosis as a predictor of non-response to treatment [6]. Our cohort, which included only those with advanced liver disease, had a similar overall SVR rate as the one reported among patients without cirrhosis in the VALENCE study (90.12% vs 91% SVR rate).

In June of 2016, sofosbuvir/ledipasvir (SOF/LED) was introduced in Georgia [2] and the treatment guidelines were modified to include this combination DAA, resulting in little to no PEG IFN use for treatment. The Georgia treatment guidelines differ from those of the American Association for the Study of Liver Diseases (AASLD) or The European Association for the Study of the Liver (EASL), providing a unique opportunity to observe population-based outcomes with alternative HCV treatment regimens. These results can inform clinicians and policy makers in countries with a large proportion or burden of HCV genotype 3 infection that may not have access to DAAs or combinations of DAAs that are available in high-income countries of North America and Western Europe.

## Limitations

This is a short report of a study limited to HCV infected genotype 3 patients with advanced liver disease treated during the first year of National HCV Elimination Program which was launched in 2015. Until 2016 HCV infected individuals with low fibrosis level were not eligible to be enrolled. The treatment outcomes were limited to two antiviral regimens: SOF/PEG/RBV and SOF/RBV. Interferon-free regimen (SOF/RIBA for 24 weeks) was used for patients with contraindication of IFN therapy, including mental illness. National HCV elimination program was not collecting data about IFN ineligibility; accordingly, we couldn’t analyze impact of comorbidities on treatment outcome. Another limitation is that we have not adjusted for the previous treatment history because at the beginning of the program this information was not entered into the elimination program database. This information is available for the patients enrolled after 2016, when program database was updated and several variables added.

It is hoped that pan-genotypic regimens will soon be introduced in Georgia, presenting the opportunity to greatly simplify testing and treatment regimens, supporting the realization of HCV Elimination in Georgia by 2020 [20].

In conclusion, high SVR rates can be achieved among patients with HCV genotype 3 and advanced liver fibrosis, particularly among those treated with a 12-week SOF/PEG/RBV regimen. This may inform treatment for HCV infected patients in countries with limited access to newer DAAs.

## Data Availability

The datasets used and/or analysed during the current study are available from the corresponding author on reasonable request.

## Abbreviations

HCV: Hepatitis C virus
WHO: World Health Organization
DAAs: Directly acting antivirals
SVR: Sustained viral response
SOF: Sofosbuvir
RBV: Ribavirin
PEG: Pegylated
INF: Interferon
aOR: Adjusted odds ratio
LED: Ledipasvir
AASLD: American Association for the Study of Liver Diseases
EASL: European Association for the Study of the Liver
